# ‘Just a delirium’: a qualitative study of care home managers’ perspectives on barriers to delirium recognition and management in UK care homes

**DOI:** 10.1093/ageing/afag056

**Published:** 2026-03-18

**Authors:** Olivia J Russell, Kamar E Ameen-Ali, Angela C Flynn, Sarah Barnes, Jennifer Ferguson, Andrew Divers, Dorothy Newbury-Birch, Judith Eberhardt, John S Young, Mani Santhana Krishnan, Ahmad A Khundakar

**Affiliations:** Population Health Sciences Institute, Newcastle University Faculty of Medical Sciences, Newcastle upon Tyne, NE2 4AX, England, UK; School of Health and Life Sciences, Teesside University, Middlesbrough, TS1 3BX, England, UK; School of Health and Life Sciences, Teesside University, Middlesbrough, TS1 3BX, England, UK; School of Population Health, RCSI University of Medicine and Health Sciences, Dublin 2, D02 YN77, Ireland; Sheffield Centre for Health and Related Research, University of Sheffield, Sheffield, S1 4DA, England, UK; School of Social Sciences, Humanities & Law, Teesside University, Middlesbrough, TS1 3BX, England, UK; School of Social Sciences, Humanities & Law, Teesside University, Middlesbrough, TS1 3BX, England, UK; School of Social Sciences, Humanities & Law, Teesside University, Middlesbrough, TS1 3BX, England, UK; School of Social Sciences, Humanities & Law, Teesside University, Middlesbrough, TS1 3BX, England, UK; School of Health and Life Sciences, Teesside University, Middlesbrough, TS1 3BX, England, UK; Lustrum Vale, Tees Esk and Wear Valleys NHS Foundation Trust, Durham Road, Stockton-on-Tees, TS19 0EA, England, UK; School of Health and Life Sciences, Teesside University, Middlesbrough, TS1 3BX, England, UK; Translational and Clinical Research Institute, Faculty of Medical Sciences, Newcastle University, Newcastle upon Tyne, NE2 4HH, England, UK

**Keywords:** delirium, care homes, qualitative study, thematic analysis, older people

## Abstract

Delirium is a common but frequently under-recognised neuropsychiatric syndrome in care homes, especially among residents living with dementia. Delirium is associated with substantial morbidity, mortality and preventable healthcare complications, yet evidence on how it is recognised and managed in residential care remains limited. This qualitative study explored care home managers’ perspectives on delirium care within privately owned care homes in a region of England, UK, and data analysed thematically to identify key challenges and opportunities for improvement. Three overarching themes were identified:

(1) Recognising and Responding to Delirium described how detection often relied on staff familiarity with residents’ usual behaviour, with limited use of formal assessment tools and frequent difficulty distinguishing delirium from dementia, particularly in hypoactive presentations.
(2) Systemic Barriers to Quality Care highlighted the impact of chronic underfunding, fragmented communication at hospital discharge, unclear clinical responsibility and limited access to training and clinical support.
(3) Collaborative Care and Support Networks captured the role of families and external professionals in delirium management, with variable communication, inconsistent validation of care home concerns and challenges coordinating care across services.

In response to these challenges, we recommend co-produced delirium education for care staff, clearer clinical pathways and stronger interprofessional collaboration to ensure that delirium is recognised and treated as a potentially life-threatening medical emergency.

## Key Points

- Delirium is common but often under-recognised in care homes, especially among residents with dementia.
- Managers report reliance on staff intuition rather than formal assessment tools.
- Systemic issues, such as underfunding, fragmented care and limited training, hinder effective delirium management.
- Coproduced education and clearer clinical pathways are needed to improve recognition and response.

## Introduction

Delirium is an acute, fluctuating neurocognitive disorder characterised by disturbed attention, awareness and cognition, arising from medical conditions, substance effects or multiple precipitating factors [1, 2]. Early recognition and management of delirium are critical, as timely diagnosis improves recovery and reduces complications, while delayed detection increases morbidity, institutionalisation and mortality [3–6]. The clinical presentation of delirium is highly variable, encompassing symptoms such as disorientation, inattention, hallucinations and profound disturbances of the sleep–wake cycle [7]. This wide range of presenting features is compounded by the motor subtypes (hyperactive, hypoactive and mixed) of delirium, with the subtle signs of hypoactive delirium often evading detection [8]. Diagnostic complexity is a particular issue in delirium superimposed on dementia, where symptom overlap can lead to poorer recovery, accelerated cognitive decline and increased long-term care dependency [9, 10].

### Delirium in care home settings

The prevalence of delirium in long-term care facilities has been estimated between 10% and 40% [11], where it is associated with elevated mortality, functional deterioration and frequent hospital admissions [12]. Despite its prevalence and impact, delirium remains under-recognised and under-prioritised in residential care [13]. Delirium in this setting also remains less understood than in acute care, and an understanding of specialist expertise or diagnostic tools in this context remains limited [14, 15]. Care home managers are pivotal in shaping organisational practice, yet while their perspectives are documented in dementia and end-of-life care [16–18], their role in understanding and managing delirium remains unexplored.

### Study aim

This study examines how UK care home managers recognise and manage delirium, to identify key barriers and inform the development of practical strategies to improve care quality, staff education and interprofessional collaboration.

## Methods

### Study design and epistemological position

This qualitative study used online focus groups to explore how care home managers understand, recognise and respond to delirium in residential settings. The study is reported in accordance with the Consolidated Criteria for Reporting Qualitative Research (COREQ) [19] Full details of the COREQ checklist are provided in Appendix 1 in the Supplementary Data section. Focus groups were chosen to facilitate interactive discussion and collective reflection on shared organisational challenges [20]. Participants were recruited using purposive sampling and were identified and recruited by local authority commissioners for care homes, who had permission to contact managers by email on behalf of the research team. Online delivery via Microsoft Teams enabled participation from geographically dispersed care home managers, for whom face-to-face attendance would have been impractical due to workload pressures and scheduling constraints. Patient and public involvement informed study development, with a regional care-home and dementia advisory group reviewing the study plan and topic guide prior to data collection.

### Research team and reflexivity

The focus groups were co-facilitated by the lead researcher, a male senior lecturer in biomedical science at the time of the study, with a research background in neuropharmacology and neuropathology. The lead researcher holds a PhD in Applied Neuroscience and was completing a Master of Public Health degree at the time of the study. Although not a practising clinician, he has a lived-experience interest in delirium. A female postgraduate research assistant supported facilitation, technical management and field notes. The lead researcher had a prior professional acquaintance with one participant, known through academic and networking events; this was managed reflexively through journaling and critical questioning during analysis. No other participants knew any members of the research team. Participants received a written Participant Information Sheet, which outlined the study’s aim to improve delirium care in care homes based on managerial insights and provided written informed consent. Data analysis was supported by an experienced qualitative researcher, with themes reviewed by a multidisciplinary team to ensure rigour.

### Participants and data collection

Ethical approval was obtained from a University Research Ethics Committee (Ref: 23777). Participants received a written Participant Information Sheet, which outlined the study’s aim to improve delirium care in care homes based on managerial insights and provided written informed consent electronically. Confidentiality and the right to withdraw within 4 weeks were assured. Five focus groups were initially planned according to local authority boundaries. After obtaining consent, one scheduled group had no attendees and another was cancelled due to insufficient participants. One further consented participant was unable to attend. This resulted in three conducted focus groups with 15 participants. A topic guide was used to explore delirium recognition and management, perceived barriers, staff training and engagement with families and professionals. Full details of the topic guide are provided in Appendix 2 in the Supplementary Data section. No nonparticipants were present. Each session lasted ~80 minutes (range 60–90). Discussions were audio-recorded using the Microsoft Teams platform. Automated transcriptions were generated, manually checked for accuracy against the recordings by the research team, and de-identified by replacing all names and care home identifiers with region-specific participant codes (e.g. DA01, ST02). Field notes were taken during and after the sessions to capture contextual observations and initial reflections.

### Data analysis

Data were analysed using reflexive thematic analysis, following the guidance of Braun and Clarke [21]. The process was iterative and abductive, involving movement between the data, emerging codes, developing themes and relevant literature. Analysis was led by the lead researcher. To ensure analytical credibility, a subset of transcripts was independently coded by an experienced qualitative researcher and initial coding frameworks and emerging themes were discussed and refined in meetings between the lead researcher and individual team members. Data were managed using NVivo software (v.15.2.0). The analytical process involved: (i) repeated reading of transcripts for familiarisation; (ii) systematic generation of initial codes across the full dataset; (iii) collation of codes into potential themes; (iv) iterative review and refinement of themes; and (v) defining and naming the final themes, which were reviewed and approved by members of the wider multidisciplinary team.

### Rigour and trustworthiness

Rigour was ensured through several strategies: reflexive practice, triangulation of analysts through independent coding and discussion of interpretations and the use of field notes to inform contextual understanding of the data. Member checking (returning transcripts to participants) was not conducted due to the de-identified, pooled nature of the focus group data and managerial time constraints, but the analytical process involved discussion and review of interpretations between the lead researcher and other team members. Recurring patterned meanings across the three focus groups suggested thematic saturation appropriate for a focused exploratory study, given the homogeneity of the sample and study aims.

## Results

### Sample characteristics

Fourteen care home managers and one deputy care home manager participated. Participants were predominantly female (93%), and all identified as White. Most were aged between 30 and 59 years, with varied educational backgrounds, and managerial experience ranging from <1 to >20 years. Almost half had undertaken previous delirium training (Table 1). This variation in prior knowledge likely contributed to a range of perspectives on the ease of recognition and management discussed in the focus groups.

**Table 1:** Participant demographics (*n* = 15). *% rounded to whole number

Variable	Category (area code)	*n* (%)^*^
**Region**	DA	5 (33)
	ST	4 (27)
	MI	6 (40)
**Age group**	18–29	0 (0)
	30–39	5 (33)
	40–49	3 (20)
	50–59	5 (33)
	60+	2 (13)
**Gender**	Female	14 (93)
	Male	1 (7)
**Ethnicity**	White	15 (100)
**Educational attainment**	Further education (A-Levels, NVQ or equivalent)	7 (47)
	Undergraduate degree	3 (20)
	Postgraduate degree	5 (33)
	<1 years	1 (7)
	1–5 years	5 (33)
	6–10 years	4 (27)
	11–15 years	1 (7)
	16–20 years	2 (13)
	20+ years	2 (13)
**Type of care home**	Residential	6 (40)
	Nursing	2 (13)
	Dementia-specific	4 (27)
	Other (e.g. mental health, learning difficulties)	3 (20)
**Care home size (residents)**	11–25	1 (7)
	26–50	3 (20)
	51–75	10 (67)
	76+	1 (7)
**Previous delirium training**	Yes	7 (47)
	No	8 (53)

A-Levels, advanced levels; NVQ, National Vocational Qualification.

### Focus group findings

Three virtual focus groups were conducted via Microsoft Teams between December 2024 and January 2025, each lasting ~80 minutes (range 60–90 minutes). Thematic analysis identified three overarching themes shaping experiences of delirium management in care homes.

### Theme 1: Recognising and responding to delirium

Participants described delirium as a complex and unpredictable condition, often marked by sudden changes in behaviour and functioning. Recognition was described as strongly dependent on staff familiarity with residents’ baseline cognition and behaviour:

*‘It could be a sudden change within hours or days and it can change the whole presentation – the whole person, who they are’.* (DA02)

Managers reported that residents were often discharged from the hospital with undiagnosed or unresolved delirium:

*‘You’re going off what the hospital are telling you…. They probably don’t know them very well… and they’re completely wrongly diagnosed (with dementia), when it’s a delirium’.* (MI04)

According to participants, when recognised, hyperactive delirium drew quicker responses, while hypoactive forms frequently went unnoticed:

*‘We had two paramedics who had never seen it before and they were convinced that he was dead. I was like, “He's not. It's hypoactive delirium.” And they'd never heard of it. Never heard of it, never seen it. And they were convinced’.* (ST01)

Participants reported that use of structured screening tools varied, with some participants familiar with structured delirium assessments such as the Four ‘A’s Test (4AT) [22], while other managers acknowledged that delirium detection was largely informal and reliant on experience:

*‘It’s not something we look at. No, there isn’t a tool, per se’.* (DA02)

Participants endorsed person-centred strategies, rather than medication alone:

*‘The first port of call shouldn’t be to just add another medication, because… you don’t know whether it will have a positive or negative effect’* (ST04).

Despite this preference, managers reported limited involvement in prescribing decisions, describing how medications were prescribed by GPs and dispensed via pharmacies without prior communication with care-home staff:

*‘Medication just turned up for her. They didn’t tell us anything’.* (ST01)

In response, managers emphasised defensive documentation of residents’ clinical presentation and care decisions to ensure accountability:

*‘All I can say as a manager is “just keep documenting it.”’* (MI04)

### Theme 2: Systemic barriers to quality care

Participants identified chronic underfunding, fragmented communication and inconsistent clinical input as central barriers to safe and timely delirium care.

They described chronic underfunding as most evident in the mismatch between available resources and escalating care expectations:

*‘We’re all funded at a very low amount, but the expectancy of the care... that we have to provide with that small amount is, well, it doesn’t match’.* (DA03)

This was particularly evident, they reported, when staff were required to escort residents to the hospital, often at the expense of staffing levels within the home:

*‘We have to fund that ourselves. And so we are paying for extra staffing when someone needs that support’.* (DA03)

Managers described fragmented communication, especially at hospital discharge, posing a significant risk, with residents returning to care homes with incomplete or delayed documentation:

*‘Ours would be hospital discharges, which is our biggest... getting somebody else’s documentation, which we’ve had on numerous occasions’.* (MI01)

Participants reported hearing dismissive language from some healthcare professionals when raising concerns about delirium:

*‘The phrase, “it’s just a delirium,” is one that’s really, really winding me up more than anything at the minute, because it’s not just a thing’.* (ST01)

Inconsistent clinical responsibility further compounded these challenges, with managers describing how they felt caught between services:

*‘We’re stuck between the GP and ICLS [Intensive Community Liaison Service: a local service mental health team], the GP says, “Well, it’s not for the GPs, it’s for the mental health,” and then you’re stuck’.* (MI06)

As a result, they reported that responsibility for recognising and escalating delirium was often placed on staff without sufficient training:

*‘The carers don’t have the insight of a nurse, they’re not clinically trained to that level, but they’re expected to assess residents and make decisions to escalate care on very little training…’* (DA05).

Participants described the impact of supporting residents through prolonged and complex delirium episodes on staff wellbeing:

*‘We’ve got staff that are actually scarred by this lady now’.* (ST01)

Training was widely described by participants as insufficient and overly reliant on e-learning:

*‘It’s just e-learning... To me, it doesn’t stick’.* (ST01).

Managers proposed the introduction of delirium champions to support training and continuity:

*‘I think for key people… create a delirium champion… so they can deliver that training to the rest of the team’.* (DA02)

### Theme 3: Collaborative care and support networks

Managers reported that effective delirium care relied on strong internal teamwork, shared vigilance and routine communication. They described collective monitoring of residents and shared understanding of baseline functioning as critical to early recognition.

A key element of this internal collaboration was described by participants as ‘prework’, involving gathering evidence and excluding reversible causes before contacting external services:

*‘Doing everything we can as pre-work, ruling out infection and everything else... before you do the referral’.* (ST02)

They reported that continuity of care and shared knowledge of residents supported confident escalation:

*‘It’s about that vigilance about... behaviours... social interactions... understanding the resident and their needs’.* (DA02)

Shared clinical tools, such as the National Early Warning Score (NEWS)2 system, were described as strengthening internal communication and confidence in clinical decisions:

*‘All the care staff know how to use it and they can put it in... it gives you the evidence there to stand your ground’.* (ST04)

Alongside internal teamwork, managers saw families as essential partners in recognising change, offering baseline knowledge and validating staff concerns:

*‘Families are very important because they know the person better than you’.* (DA02).

However, many relatives were described as finding delirium distressing and confusing, sometimes leading to misplaced frustration with staff:

*‘They think once you’ve finished a course of antibiotics, it should be finished… why isn’t she any better?’* (ST01)

Support from families was not universal, managers reported, with many residents having no family advocates, increasing emotional and practical demands on care home staff:

*‘They may ask for staff support too. And if you can’t provide it, they then will threaten you with safeguarding’.* (DA03)

## Discussion

The study revealed a system where care home staff, despite variable formal training, develop a contextual expertise on delirium through intimate knowledge of residents. This expertise, however, is often marginalised within fragmented care pathways, leaving staff to manage complex clinical situations with limited support. This reflects a wider neglect of delirium within policy frameworks [23] and highlights a critical gap between front-line observational knowledge and formal clinical systems.

This study emphasises the critical, yet unstable, role of experiential knowledge in bridging formal clinical gaps. While some managers demonstrated practical understanding of the multifactorial nature of delirium, consistent with established models [24], this was uneven and often unsupported by training. Nevertheless, the experience of navigating fragmented services and feeling professionally marginalised was common across participants, regardless of training background. Participants commonly described challenges in delirium care as arising from external services, particularly at points of prescribing, discharge and escalation, pointing to a systemic failure of accountability and integration. This suggests that improving delirium care must move beyond training alone and focus on developing structures that validate and incorporate care home staff’s contextual insights into shared decision-making.

A key diagnostic complexity highlighted by managers was distinguishing delirium from dementia, especially posthospitalisation. This is critical given delirium’s link to poor long-term outcomes [6]. However, managers reported rarely receiving hospital screening results (e.g. 4AT scores), which can aid differential diagnosis [25, 26]. The well-documented problem of under-detecting hypoactive delirium [8, 27] was also evident in our findings and exacerbated by perceived dismissiveness from some external professionals. Managers’ reports of prolonged delirium episodes further challenge the notion of it as a transient acute issue, positioning care homes as key settings for managing its extended course.

Although there remains a paucity of qualitative studies examining care home managers’ perspectives on delirium specifically, the challenges they described resonate with findings from other professional groups. For example, hospital nurses have reported similar experiences of moral distress when managing complex delirium without adequate support [28]. More broadly, the systemic barriers to integrated care identified by our participants, such as fragmented communication and unclear clinical responsibility, mirror difficulties in implementing coordinated delirium prevention and management systems in other settings [29]. Finally, the pivotal yet constrained managerial role observed here aligns with qualitative evidence on care home managers’ experiences in dementia and end-of-life care, which similarly emphasises high responsibility coupled with limited authority and resources [17, 18].

The findings suggest significant ethical dimensions to the care of residents with delirium, including moral distress when managing complex cases without support [28, 29]. As previously, while nonpharmacological approaches were preferred [30], their implementation was hampered by systemic pressures. Similarly, relationships with families and external professionals offered opportunities and challenges, being vital for support yet potential sources of conflict [31, 32], emphasising the need for true partnership and shared understanding with care home staff.

### Future directions and proposed interventions

Building on the perspectives of care home managers, future efforts should focus on interventions that bridge the gaps identified between experiential knowledge and formal systems. First, co-produced training for care home staff should move beyond generic e-learning to become more context-sensitive, incorporating validated tools such as the 4AT alongside person-centred approaches. Second, service development must address communication failures, particularly by routinely sharing delirium screening results at hospital discharge. Finally, future research should explore models such as the Delirium Champion role [33], which may help empower care home staff within multidisciplinary teams. Further investigation of the emotional labour and ethical challenges associated with delirium care in this setting should be prioritised.

### Study limitations

The limitations of this study should be acknowledged. The sample was predominantly female, ethnically homogenous managers from one region of England, which may limit transferability. The qualitative design prevented generalisability, and voluntary recruitment may have introduced bias. The exclusion of frontline carers, residents and family members limited the breadth of perspectives captured, and findings may be influenced by the post-COVID-19 context of workforce and funding pressures. Variation in participants’ prior delirium training was not explored and, alongside their tendency to attribute challenges to external services, may reflect variable knowledge or clinical authority. Finally, participants were not asked to propose solutions; thus, the future directions outlined are research-informed priorities rather than direct participant recommendations.

## Conclusion

This study with UK care home managers highlights systemic barriers to delirium care, where staff’s observational expertise is often marginalised within fragmented systems. Sustainable improvement requires co-produced training, stronger interprofessional communication and care models that formally integrate the contextual knowledge of care home staff. Most importantly, delirium must be acknowledged as a serious, potentially life-threatening condition demanding urgent action, not dismissed as ‘just a delirium’.

## Supplementary Material

Supplementary_materials_afag056

## Research Data Transparency and Availability

The qualitative data generated and analysed in this study consist of audio recordings and verbatim transcripts from focus groups with care home managers. These data contain sensitive and potentially identifiable information, and the ethical approval for this project does not permit sharing of raw data beyond the research team. Consequently, the data are not publicly available. Enquiries regarding the study may be directed to the corresponding author.
