# Supplementary material for: ‘Just a delirium’: a qualitative study of care home managers’ perspectives on barriers to delirium recognition and management in UK care homes
Source: Age Ageing. 2026 Mar 18;55(3):afag056. doi: 10.1093/ageing/afag056 (PMC13017741; doi:10.1093/ageing/afag056)
Supplement: Supplementary_materials_afag056 [file supplementary_materials_afag056.zip › Supplementary_materials_afag056_appendix2.docx]

**“Just a Delirium”: A Qualitative Study of Care Home Managers’ Perspectives on Barriers to Delirium Recognition and Management in UK Care Homes**

**Appendix 2. Focus Group Topic Guide.**

Good [morning/afternoon] everyone. Thank you for joining us for this discussion. My name is Dr. XXXXX and I’m here with XXXXX, an MPH student who will ensure the session is being recorded accurately. We’ll be discussing your experiences and perspectives on managing delirium in care home settings. We greatly appreciate your time and insights.

We want to reassure you that this conversation is confidential. No names or identifying details will be included in our reporting and all information will be handled respectfully and securely. Our aim is to better understand your experiences so that we can support improved care practices.

This discussion will take approximately 60 minutes. If at any point you need clarification on a question or would prefer not to answer, please feel free to let us know.

**1. Current Role Experience**

- Question: Can you describe your experience of working in this current role?
  - Follow-up: What are some notable challenges or successes you've encountered?

**2.** **Challenges in Care Home**

- Question: What are the main challenges your care home faces with resident care or health management?

**3.** **Understanding of Delirium**

- Question: Could you share your understanding of what delirium is?
  - Prompt: Often described as an acute state of confusion that changes in severity.
    - How does this align with or differ from your understanding?
  - Follow-up: Do you or your staff refer to this condition using any other terms?

**4.** **Managing Delirium in Your Care Home**

- Question: Can you describe your experiences with managing delirium in your care home?
  - Prompts:
    - What strategies do you currently use to prevent and manage delirium?
    - What actions do you take when you suspect a resident has delirium?
    - How, if at all, do you involve residents' families in managing delirium?
    - Are there any specific challenges in managing delirium?

**5. Documentation of Delirium**

- Question: Do you document episodes of delirium?
  - Prompt: If so, how do you document these episodes?

**6. Training on Delirium**

- Question: Have you or your staff received training or educational programmes on delirium?
  - Prompts:
    - What aspects of these programs did you find effective or useful?
    - What resources or tools have been helpful in managing delirium?
    - What additional support or resources do you think would help improve delirium care in your facility?

**7. Assessing Management Effectiveness**

- Question: Do you have a process to assess the effectiveness of your delirium management strategies?
  - Follow-up: How do you evaluate whether your approaches are working?

**8. Additional Thoughts**

- Question: Is there anything I haven’t asked that you think is relevant to this discussion?

Thank you again for your participation and for sharing your experiences. Your insights are invaluable in helping us understand and improve care practices related to delirium management.
